# Functional Outcome of Forearm Fractures Managed With Screw Nails vs. Dynamic Compression Plates: A Prospective Study

**DOI:** 10.7759/cureus.67575

**Published:** 2024-08-23

**Authors:** Pallav P Agrawal, Sushil Mankar, Vismay V Harkare, Rahul H Sakhare, Nikhil Halmare

**Affiliations:** 1 Orthopedics and Traumatology, N. K. P. Salve Institute of Medical Sciences & Research Centre And Lata Mangeshkar Hospital, Nagpur, IND; 2 Orthopedics and Traumatology, Government Medical College and Hospital, Gondia, Gondia, IND

**Keywords:** grace and eversmann score, absolute stability, dynamic compression plate, screw nail, both bone forearm fracture

## Abstract

Background

Fractures of the forearm are very frequently encountered in day-to-day practice. These fractures have a bimodal age distribution. The forearm fractures are considered intra-articular and need absolute stability for adequate healing. The current treatment modalities include using intramedullary devices such as a square nail, locking intramedullary nail, or using a plate for fixation. In this study, we aim to determine the functional outcome of forearm fractures managed with a screw nail used as an intramedullary device as compared to a locking plate using the Grace-Eversmann criteria.

Methodology

Patients with forearm fractures were divided into two groups and treated with a screw nail and a dynamic compression plate. Patients were followed up at one month, three months, six months, and one year postoperatively and assessment was done using the Grace-Eversmann criteria.

Results

The study included a total of 30 subjects, ranging in age from 18 to 65. The majority of the patients had encountered a road traffic accident, following which they incurred a forearm fracture. Grace-Eversmann criteria was used for these patients at follow-up, and a total of 13 patients (86.6%) had good to excellent scores, which was similar when compared to the plate osteosynthesis group (86.6%). A significant difference in the amount of blood loss was noted in the screw nail osteosynthesis group as compared to the plate osteosynthesis group (p<0.05).

Conclusions

Though a dynamic compression plate is considered a standard method for fixation of the forearm fractures, the use of an intramedullary screw nail as a fixation device gives a similar result with excellent functional outcomes (Grace-Eversmann criteria). It also gives an added benefit of reduced blood loss and preservation of fracture biology.

## Introduction

Fractures of the forearm are very frequently witnessed in a setting of trauma. They have a bimodal age distribution and are commonly seen in children and the elderly [1]. The forearm plays a vital role in all daily activities, thus contributing greatly to hand versatility [2]. Achieving rotational stability and regaining and maintaining the length of the forearm is the main aim of every orthopedic surgeon treating the fracture [3]. The radius and ulna shaft fracture is considered intra-articular, which thus requires anatomical reduction to prevent impairment of hand function and positioning [4]. Surgery for fracture fixation using an open reduction approach is the mainstay of treatment. Using a dynamic compression plate (DCP) gives a more biological outcome and is known to achieve a high union rate. Its use is also associated with long incisions, more surgical time, and disturbance of the facture hematoma [3].

Segmental configuration of the forearm fracture is quite frequent and may prove to be a challenge for management. Determining factors in the stability and reduction of the fracture are the muscle strength exerting a deforming force and also depend on the presence or absence of injury to the intra-osseous membrane [5].

The use of intramedullary devices for the fracture of the forearm fixation is frequent in the pediatric age group, but similar results have not been achieved in the adult population as they fail to provide rotational and linear stability [6].

The advent of locking intramedullary nails for forearm fracture management provides a distinct advantage with the prevention of shortening in comminuted and segmental diaphyseal fractures of the forearm but is usually accompanied by a risk of injury to the posterior interosseous nerve, and the technique is difficult to master. A high rate of non-union has been observed with intramedullary nailing with Rush pins, Kirschner wires, and Steinman pins. However, unlocked nails do not adequately control rotation [3].

An intramedullary screw nail is a nail with a circular cross-section, one end being bevel and the other end being threaded. The fracture can be reduced with the distal bevel part of the nail as it helps engage the subchondral bone, thus imparting stability. It works on the principle of fixation at three points of contact and helps to maintain the radial bow. The screw end of the nail, after being buried into the metaphyseal region of the bone imparts stability and prevents migration [3]. Relative stability is provided to the fractured bone which aids in fracture healing [7].

The current study is performed with the aim to compare the outcome of the forearm shaft fractures managed with conventional plate osteosynthesis and screw nail osteosynthesis.

## Materials and methods

A prospective study was conducted in the Department of Orthopedics, N. K. P. Salve Institute of Medical Sciences & Research Centre, and Lata Mangeshkar Hospital, Nagpur. Institutional Ethics Committee approval was taken (approval number: 75/2019). A sample size of 30 patients was determined based on the study done by Jie-Jia Mi et al. [8] and using the formula to determine the sample size:

n = [z(Alpha) + z(Beta)] x (Standard Deviation)^2^ divided by [X1-X2]^2^ 

X1 is the mean time for union in the plate fixation group, and X2 is the mean time for union in the nail fixation group. Alpha is the significance level of the study, and "1- Beta" is the power of the study. 

The study included 30 subjects, aged 18 years and above, who had both bone forearm fractures and isolated radial or ulnar shaft fractures, and who were willing to undergo surgery. Random allocation using an odd and even randomization method was done, and patients were divided into two groups, with one group being treated with an intramedullary screw nail and the other by a dynamic compression plate from December 2019 to October 2021. Patients were followed up at one month, three months, six months, and one year. Patients with Gustilo-Anderson Grade 3B or 3C open fractures, patients with fractures of the radio-ulnar joint with dislocation, patients with bilateral fractures, and patients not willing for surgery were excluded. 

Grace-Eversmann criteria was used to determine the outcome, and it was measured at the follow-ups. It comprises assessing the fracture's union and comparing the ratio of pronation-supination to the uninjured arm (Table 1) [9]. 

**Table 1:** Grace-Eversmann criteria

	Union	Pronation supination comparison ration with the uninjured arm
Excellent	+	90-100%
Good	+	80-89%
Acceptable	+	60-79%
Unacceptable	-	<60%

Pre-op workup

An elaborative history was obtained from the subjects, focusing on injury mechanisms, other injuries that might be associated, other medical illnesses, and status at the pre-injury time. A complete patient evaluation was done after examining for systemic or generalized conditions. Examination of the fractured limb with an assessment of soft tissue and skin was also done. Hematological investigations were conducted and studied. The general condition was built up, the patients were optimized for surgery, and appropriate temporary stabilization of the fracture using an above-elbow slab was done. Fitness for surgery was obtained from the anesthesia team. A standard anteroposterior and lateral view of the forearm with the elbow and wrist joint was taken, and fracture anatomy was studied.

Pre-op assessment and planning

All the patients were prepared for surgery. Patients signed an informed consent. All patients in the study underwent routine preoperative preparation. They were administered similar antibiotics at the start of the surgery. A tourniquet was applied over the arm and inflated after exsanguinating the extremity to the appropriate level to achieve a bloodless field in both groups.

Operative technique

Nail Insertion for Radius

**Figure 1:**
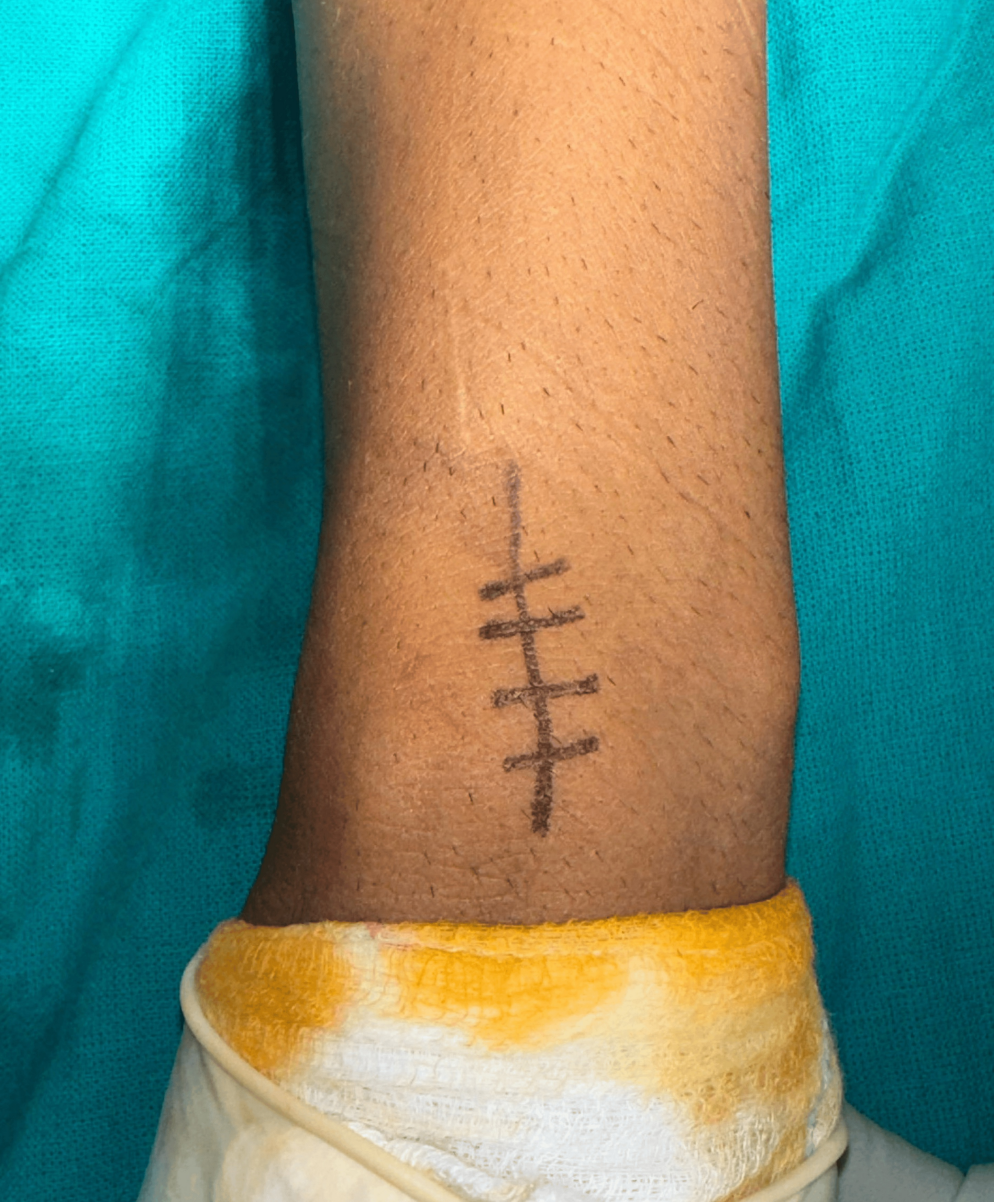
Incision and entry point for radius nail on dorsal aspect.

The subjects were taken in a supine position with the arm abducted and the elbow extended. The incision is taken on the wrist over the dorsal aspect of approximate size 2 cm, lateral to the lister’s tubercle (Figure 1). Entry is made with the help of bone awl under the c-arm guidance. An appropriately sized nail was taken and slightly pre-bent to match the contour of the bone and inserted through the incision. The nail end was buried to avoid irritation of the extensor tendons.

Nail Insertion for Ulna

Patients were taken in a supine position with the abduction of the shoulder and 90-degree flexion of the elbow joint. The reduction was achieved with traction and manipulation. The fracture was approached through the tip of the olecranon process under c-arm guidance, and bone awl was used to make entry into the medullary canal. A screw nail of suitable diameter and length was passed through the incision. The nail end was buried inside the olecranon.

Radius-Ulnar Plate Osteosynthesis

**Figure 2:**
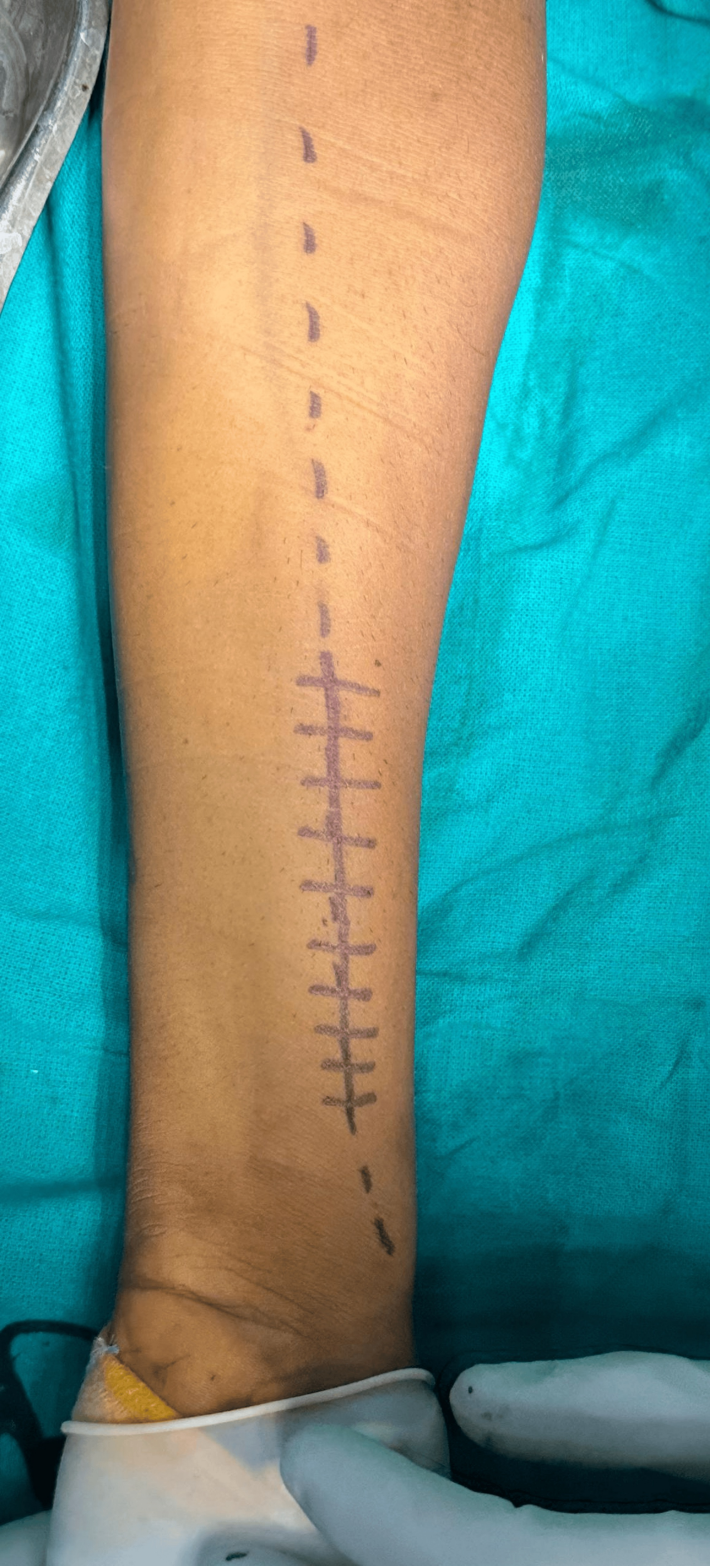
Image showing the incision for plate osteosynthesis in radius on volar aspect.

Internal fixation after open reduction of the radius was done via the routine approaches for radius (Henry’s approach for volar exposure and Thompson's approach for dorsal exposure) (Figure 2), and a universal ulnar approach was used for the open reduction and fixation of the ulnar fracture. A tourniquet was applied throughout the surgery, and the patient was taken in supine position with the arm abducted. A dynamic compression plate was used in all the cases for both the bones.

Postoperative management

Postoperatively, patients in the nail group were given immobilization with the above elbow slab for three weeks, following which they were started on physiotherapy for elbow range of motion and wrist range of motion. Patients in the plate group were mobilized as and when tolerated by them.

All the patients were started on a similar anti-biotic regimen of intravenous antibiotics for three days postoperatively, followed by oral anti-biotics till suture removal. Dressing of the suture site was done on days two and six for all the patients.

Follow-up

All the patients in both groups were followed up at one, three, six months, and one year. Radiographs were obtained during follow-up along with a clinical evaluation using the Grace-Eversmann criteria. Radiographs were obtained during follow-ups, and elbow and wrist range of motion was recorded (Figure 3-11).

**Figure 3:**
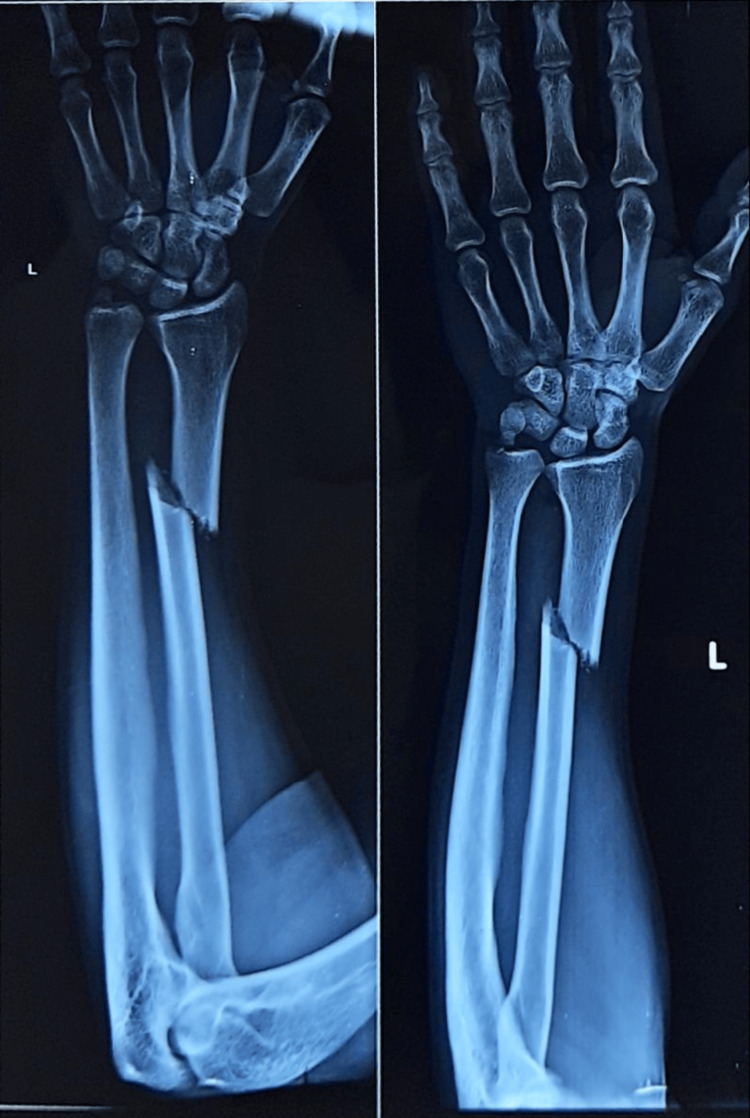
Pre-operative X-ray showing fracture shaft radius (nail osteosynthesis group).

**Figure 4:**
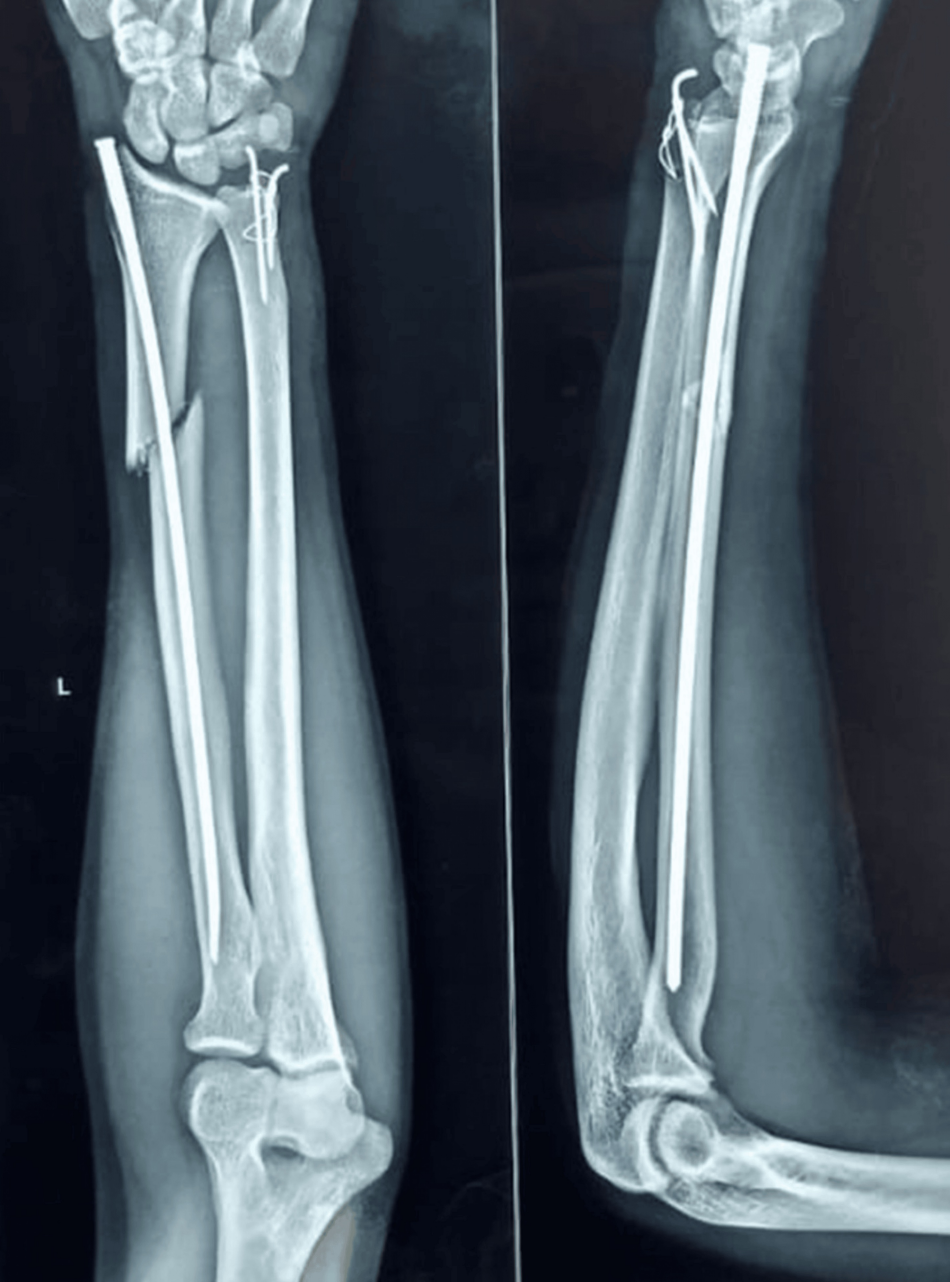
Immediate post-op X-ray of fracture shaft radius managed with screw nail osteosynthesis with tension band wiring (TBW) for ulnar styloid.

**Figure 5:**
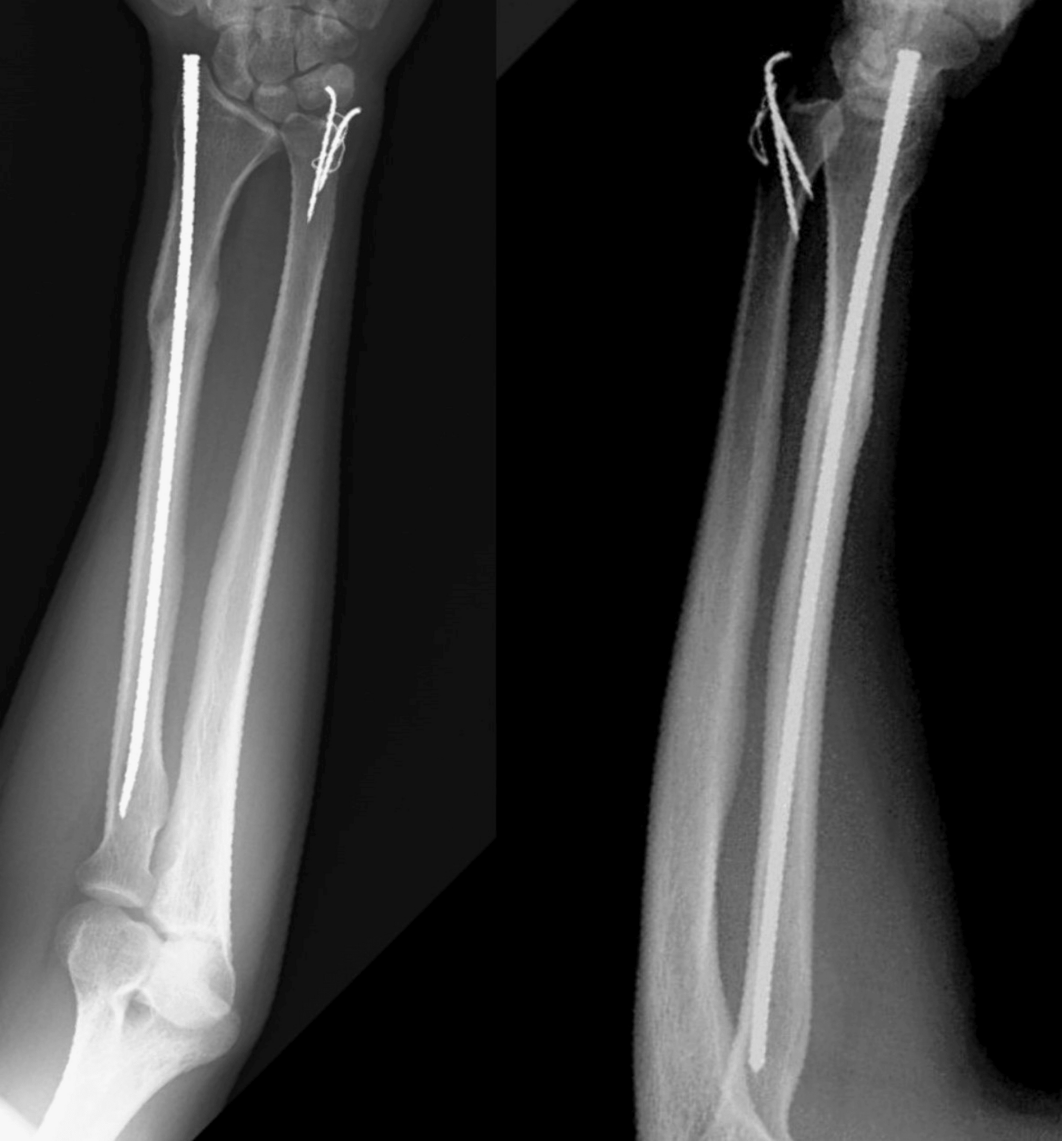
Postoperative X-ray of fracture shaft radius managed with intramedullary screw nail (one year follow-up) with TBW done for ulnar styloid fracture. TBW:  tension band wiring

**Figure 6:**
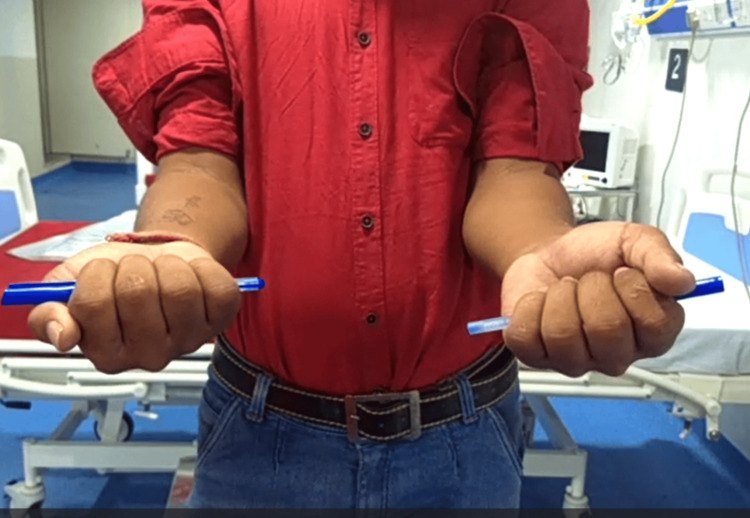
Clinical photo of forearm in supination (left-side operated).

**Figure 7:**
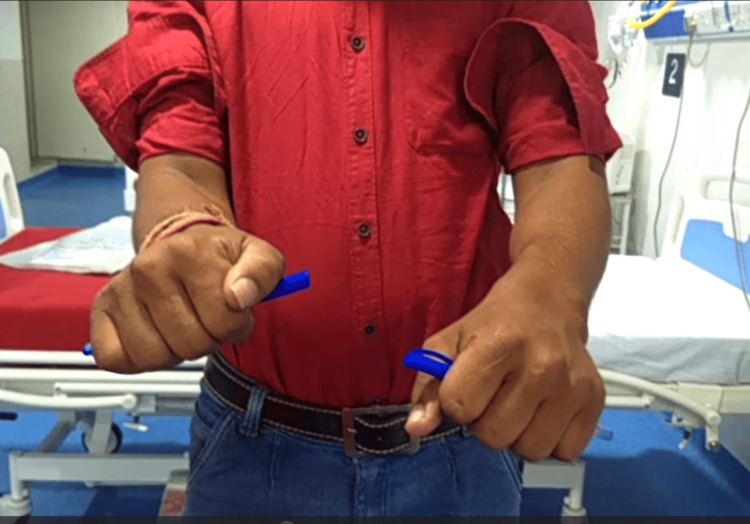
Clinical photo of forearm in pronation (left-side operated).

**Figure 8:**
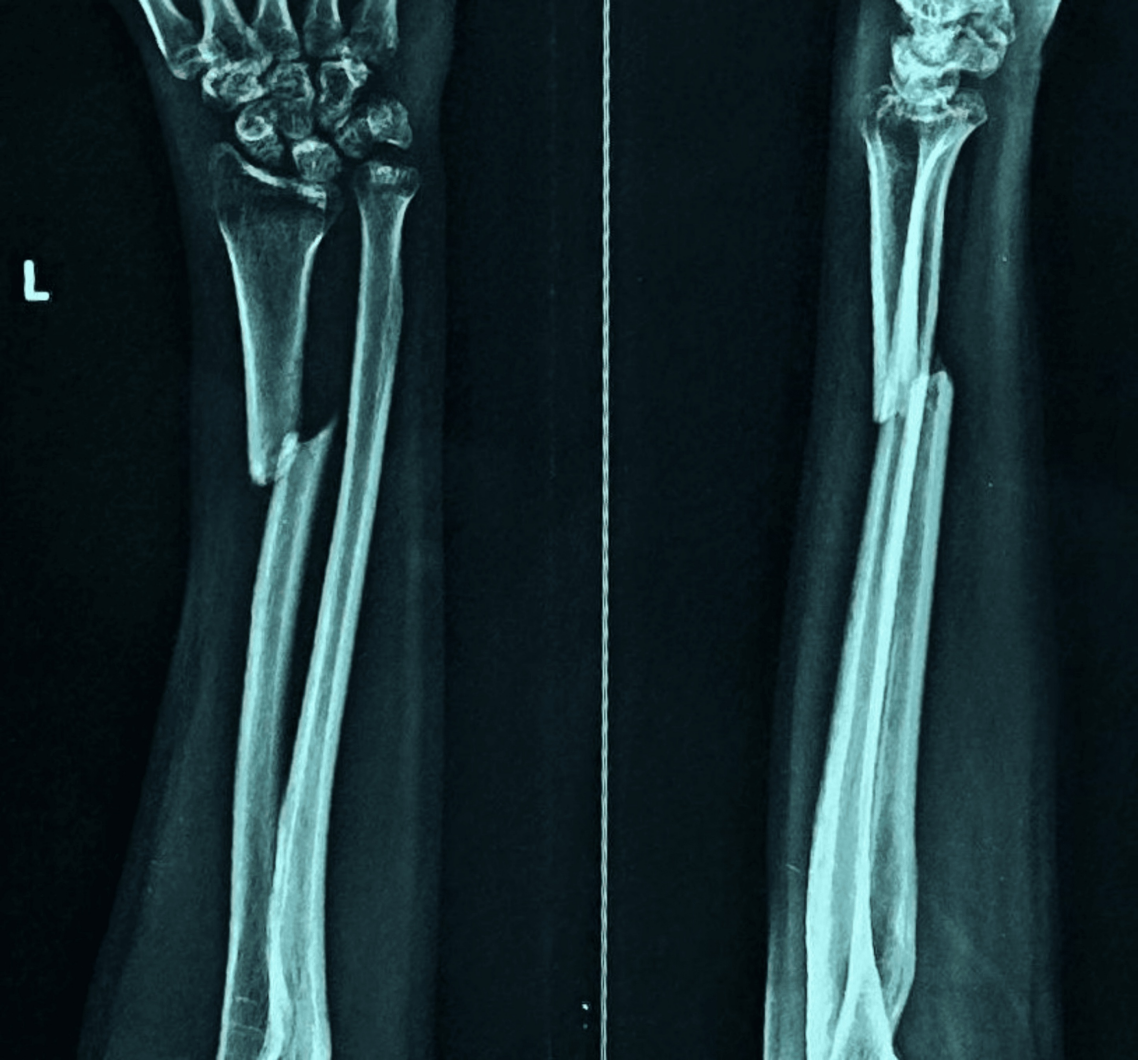
Pre-operative X-ray showing radial shaft fracture (plate osteosynthesis group).

**Figure 9:**
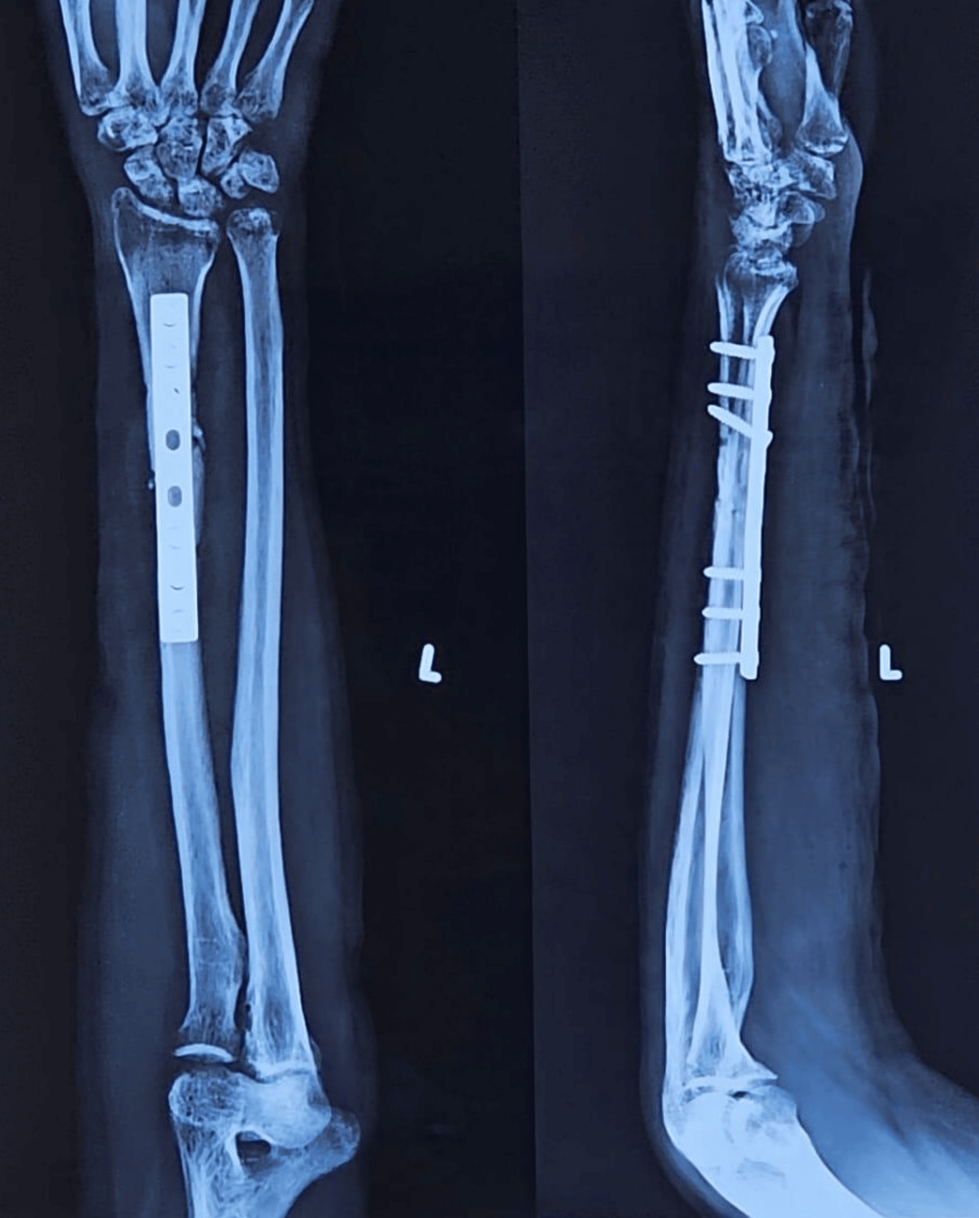
Postoperative X-ray of fracture radial shaft managed with dynamic compression plate.

**Figure 10:**
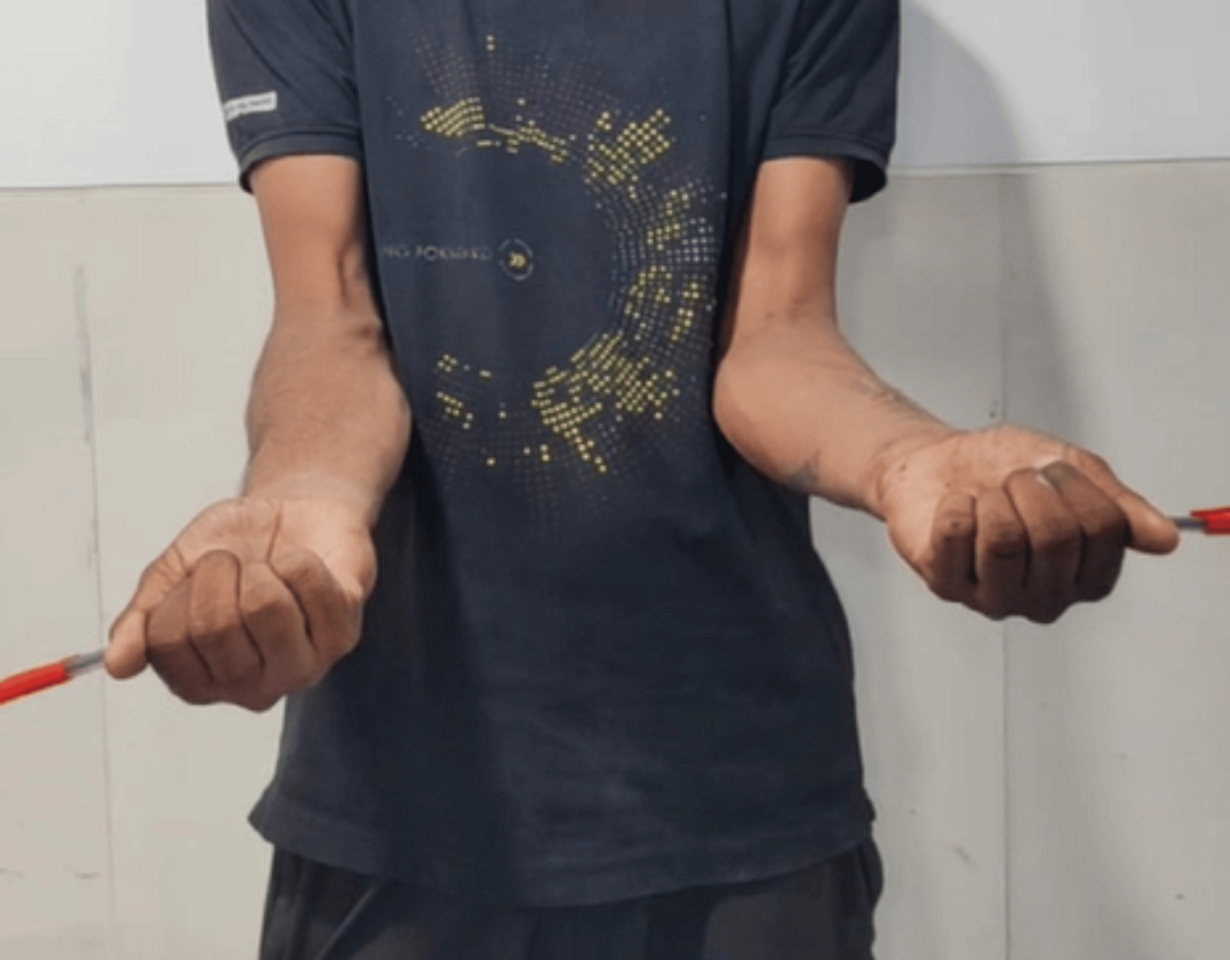
Clinical photo of forearm in supination (left-side operated).

**Figure 11:**
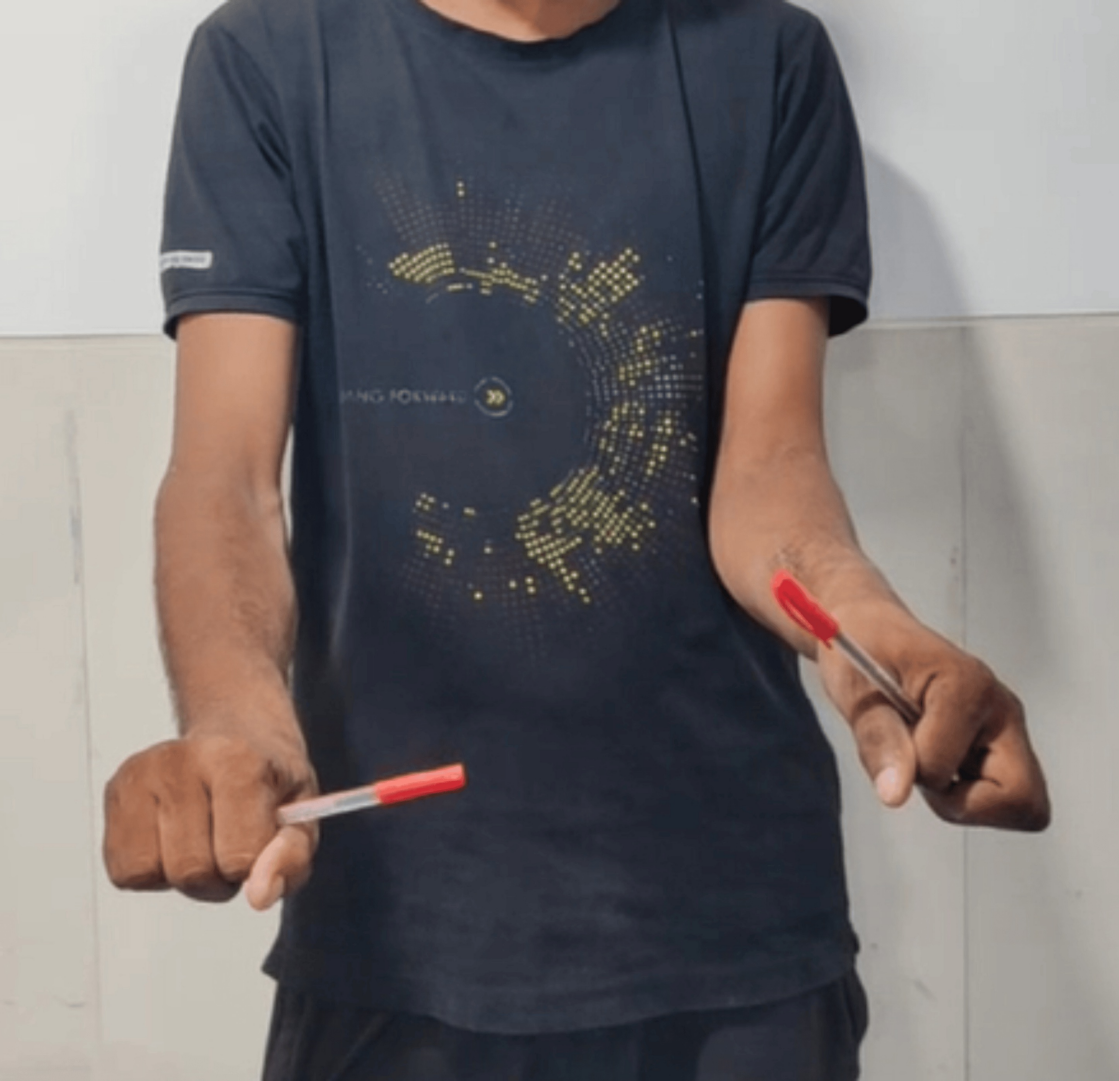
Clinical photo of forearm in pronation (left-side operated).

Stastical analysis

Patients were evaluated at the follow-ups with X-rays and the Grace-Eversmann criteria. They were evaluated based on the time of surgery, blood loss during the surgery, and the time to union. Data analyses were done by a statistical program, R. A chi square test was used to study the non-parametric data and a paired t-test to study the normal distribution of data. P-values less than 0.05 were considered significant.

## Results

Our current study included 30 subjects distributed amongst two groups between 18 years and 65 years. Most patients ranged between 18 and 30 years of age (46.6%). The maximum number of patients had a history of road traffic accidents (RTA) (86.6%).

Grace-Eversmann criteria was used to perform a clinical evaluation for these patients; a total of 13 patients (86.67%) had good to excellent scores in the intramedullary group, and a similar score was seen in patients managed with plate osteosynthesis at the end of one year. There was no statistically significant difference between the two groups at the end of one year (p=0.982) (Table 2).

**Table 2:** Results showing the Grace-Eversmann criteria in the nail and plate osteosynthesis group at the end of one year.

Grace-Eversmann criteria	Intramedullary nail group	Plate osteosynthesis group	P-value
Excellent	8(53.3%)	9(60%)	0.982
Good	5(33.3%)	4(26.6%)	
Acceptable	1(6.7%)	1(6.7%)	
Unacceptable	1(6.7%)	1(6.7%)	
Total	15(100%)	15(100%)	

Time to union was found to be 12-16 weeks in 66.67% (10/15) and 60% (9/15) of nail and plate osteosynthesis patients, respectively (p=0.4734). 

The total time required for the surgery in the plate osteosynthesis group was more as compared to the time required in the nail osteosynthesis group, but the difference was not significant (p>0.05). The amount of blood loss witnessed in the nail osteosynthesis group was significantly lower as compared to the plate osteosynthesis group (p<0.05) (Table 3).

**Table 3:** Table showing the results of the parameters studied.

Parameters	Plate osteosynthesis group	Screw nail osteosynthesis group	P-value
Blood loss (in ml)	203.33 ± 35.28 ml	22.33 ± 4.16 ml	0.001
Operative time (in minutes)	78 ± 13.2 minutes	54 ± 8.409 minutes	>0.05

## Discussion

Forearm fractures are common in the general population and are usually managed operatively. They can be managed conservatively with cast application but have their own set of complications, such as malunion, bayonet apposition, and compartment syndrome [10].

For good results, the fracture's anatomy must be precisely restored. Osteosynthesis using a plate after open reduction is considered an acceptable treatment modality and has good functional results, but is also associated with problems such as the opening of the fracture hematoma and increased chances of infection [11].

Intramedullary nail fixation, which acts as a central load-bearing implant, has lots of advantages, such as minimum handling of the periosteum and hematoma preservation [11]. The flexibility of rush pins imparts stability by three-point contact while maintaining the radial curve, but its end acts as a potential irritant, and a thinner pin fails to address the rotational stability. The square design of the nail, which helps to improve stability and fracture healing dramatically, has decreased the rates of non-union in these fractures, but implant migration remained a major concern while using these nails [3]. All the above complications and difficulties are tackled by the use of an intramedullary elastic screw nail, which prevents implant migration due to the screw end of the nail.

The goal of our present study was to determine the functional outcome of forearm fracture managed with an intramedullary device using screw nails as compared to open reduction internal fixation with plate osteosynthesis.

Gadegone et al. used a screw-elastic nail for internal fixation of the fractures, while other authors used conventional intramedullary nails (square nail, interlocking intramedullary nail) [3]. They noticed good to excellent results in 89% of the cases, which was similar to our results of good to excellent scores in 73.3% of individuals in the nail group based on Grace-Eversmann criteria [3]. The scores were good to excellent in the plate and nail osteosynthesis groups in 70% each in the study by Garmapalli et al. [12], 30% (nail) and 40% (plate) in the study by Ramavtar Saini et al. [4], and 82.3% (plate) and 81.3% (nail) in the study by Nazan Cevik et al. [5]. The scores suggest a similar outcome in the plate and nail osteosynthesis groups in these studies, suggesting a similar functional outcome. These results are identical to those obtained by our current study, i.e., 86.6% (nail) and 86.7% (plate) had good to excellent scores at the end of one year.

Ramavtar et al. [4] stated the surgical time to be 57.25 minutes for the nail group and 75.5 minutes for the plate group. Ozkaya et al. [13] also stated an operative time of 65 minutes for the plate osteosynthesis group and 61 minutes for the nail osteosynthesis group. The time required for the nail osteosynthesis group was less in all the studies as compared to the plate osteosynthesis group. In our current study, the surgical time was 78 minutes for the plate osteosynthesis group and 54 minutes for the nail osteosynthesis group. 

In our study with a one-year follow-up, time to union of the fracture was noted to be 13 weeks for the plate osteosynthesis group and 14 weeks for the nail osteosynthesis group. Ozkaya et al. [13] stated a lesser time to union for the nail group at 10 weeks and for the plate group at 14 weeks. Almost similar results were noted by Nazan et al. [5] in their study, with a mean time to union of 12.3 weeks and 12 weeks for the plate and nail osteosynthesis groups, respectively. The time to union for the plate and nail osteosynthesis groups was 14 weeks and 10 weeks, respectively, in the study by Lee et al. [14]. The results among various studies are similar to our current study.

In our current study, one of the 15 patients had encountered a superficial infection (6.7%) in the plate osteosynthesis group. It was managed with wound debridement and re-suturing and with the use of intravenous antibiotics. Nazan et al. [5] also reported superficial infection in two of the patients in the plate osteosynthesis group. Gadegone et al. [3] mentioned superficial infection in three patients with Grade 1 open fractures with the screw nail group. Nadeem et al. [10] reported two cases of superficial surgical site infection (5.88%) that were managed with oral antibiotics.

Non-union was noted in one patient (6.7%) in each of the groups in the current study. Delayed union was noted in three patients and mal-union in one patient. Gadegone et al. [3] also reported delayed union in two patients. Ramavtar et al. [4] mentioned delayed union in 5% of the cases and non-union in 5% of patients who both belonged to the nail group and one case of delayed union in the plating group.

Another generally noted complication seen with these kinds of fractures is olecranon bursitis, which was reported by Nadeem et al. [10] in one patient and in three patients in the nailing group by Ramavtar et al. [4]. Elbow stiffness was also reported in three patients (15%) by Ramavtar et al. [4] in the plate osteosynthesis group. The drawbacks of the current study are a limited sample size and a limited follow-up of one year.

## Conclusions

Management of the forearm fractures with plate osteosynthesis, though gold standard, and use of an elastic intramedullary screw nail provide similar results in terms of the functional outcome as measured by the Grace-Eversmann criteria, time to union, and surgical time at the end of one-year follow-up. The use of an intramedullary elastic screw nail provides an additional benefit of reduced surgical time as compared to the plate osteosynthesis group and significantly less blood loss in the nail osteosynthesis group. As the fracture hematoma is preserved, the nail osteosynthesis group can expect more biological healing. Hence, the screw nail is an ideal and affordable intramedullary implant for the fixation of forearm shaft fractures.
